# Stepwise iterative maximum likelihood clustering approach

**DOI:** 10.1186/s12859-016-1184-5

**Published:** 2016-08-24

**Authors:** Alok Sharma, Daichi Shigemizu, Keith A. Boroevich, Yosvany López, Yoichiro Kamatani, Michiaki Kubo, Tatsuhiko Tsunoda

**Affiliations:** 1 RIKEN Center for Integrative Medical Sciences, Yokohama, 230-0045 Japan; 2 CREST, JST, Yokohama, 230-0045 Japan; 3 Institute for Integrated and Intelligent Systems, Griffith University, Brisbane, Australia; 4 Medical Research Institute, Tokyo Medical and Dental University, Tokyo, 113-8510 Japan

## Abstract

**Background:**

Biological/genetic data is a complex mix of various forms or topologies which makes it quite difficult to analyze. An abundance of such data in this modern era requires the development of sophisticated statistical methods to analyze it in a reasonable amount of time. In many biological/genetic analyses, such as genome-wide association study (GWAS) analysis or multi-omics data analysis, it is required to cluster the plethora of data into sub-categories to understand the subtypes of populations, cancers or any other diseases. Traditionally, the *k*-means clustering algorithm is a dominant clustering method. This is due to its simplicity and reasonable level of accuracy. Many other clustering methods, including support vector clustering, have been developed in the past, but do not perform well with the biological data, either due to computational reasons or failure to identify clusters.

**Results:**

The proposed SIML clustering algorithm has been tested on microarray datasets and SNP datasets. It has been compared with a number of clustering algorithms. On MLL datasets, SIML achieved highest clustering accuracy and rand score on 4/9 cases; similarly on SRBCT dataset, it got for 3/5 cases; on ALL subtype it got highest clustering accuracy for 5/7 cases and highest rand score for 4/7 cases. In addition, SIML overall clustering accuracy on a 3 cluster problem using SNP data were 97.3, 94.7 and 100 %, respectively, for each of the clusters.

**Conclusions:**

In this paper, considering the nature of biological data, we proposed a maximum likelihood clustering approach using a stepwise iterative procedure. The advantage of this proposed method is that it not only uses the distance information, but also incorporate variance information for clustering. This method is able to cluster when data appeared in overlapping and complex forms. The experimental results illustrate its performance and usefulness over other clustering methods. A Matlab package of this method (SIML) is provided at the web-link http://www.riken.jp/en/research/labs/ims/med_sci_math/.

**Electronic supplementary material:**

The online version of this article (doi:10.1186/s12859-016-1184-5) contains supplementary material, which is available to authorized users.

## Background

In an unsupervised learning procedure, the class label of a training sample is not known and the aim is to partition the data into clusters. The unsupervised learning scheme uses the relationship between samples to perform partitioning. In many biological data (e.g. transcriptome data, genomic data etc.), the number of clusters and class labels are unknown. However, the distribution is sometimes known, which is usually normal. Therefore, it would be an advantage to build a technique that utilizes distance and variance information as it can track clusters with different conformations.

Over last several decades, the *k*-means clustering algorithm has been used quite significantly in partitioning the biological data. In the most recent multi-omics data analysis tools, like iCluster, and iClusterPlus [1], the underlying clustering method used was also *k*-means. Some tools in cancer research, like ConsensusCluster (CC) and CCPlus [2, 3], also utilize *k*-means as one of the common clustering algorithms. Though the *k*-means clustering algorithm has been extensively applied [4] due to its simplicity and reasonable level of accuracy, it cannot track clusters when samples of different groups are overlapping to each other (i.e., data points of adjacent groups are spread in a way that the groups partly coincide over each other). In biological data, this is sometimes the case, and thereby leads to clusters which may not be accurate. This has a significant implication in biological findings, particularly in cancer subtypes analysis, population stratification analysis in GWAS and multi-omics data analysis. In general, *k*-means has played a significant role in carrying out analysis for various types of biological data over several years. Since data complexity and quantity are increasing, it is important to develop techniques that can perform clustering by following data topologies.

In the field of unsupervised learning and clustering, several wonderful techniques have emerged. Some of the techniques are briefly summarized here as follows: 1) clustering using some criterion functions e.g. i) sum-of-squared error criterion; ii) related minimum variance criterion, iii) scattering criterion; iv) trace criterion; v) determinant criterion; and, vi) invariant criterion [5, 6]; 2) clustering using iterative optimization [7–9]; 3) hierarchical clustering [10–13]; several hierarchical-based algorithms can be found in the literature; e.g., single-linkage [14], complete-linkage [15], median-linkage [16] and so on. Single linkage (SLink) [14] merges two nearest-neighbor clusters at a time in an agglomerative hierarchical fashion. It uses Euclidean distance to measure the closeness between two clusters (if it is less than an arbitrary threshold). This method is very sensitive to data position, which sometimes creates problem by forming a cluster in a long chain (known as the chaining effect). The complete linkage (CLink) hierarchical approach [15] depends on the farthest-neighbor and reduces the effects of long chains. This technique is also sensitive to outliers. The use of average or median distance could be a way to overcome this sensitiveness. This was done in median linkage (MLink) hierarchical approach [16]; 4) clustering is also performed using Bayes classifier [17–21]; 5) clustering iterative maximum likelihood [22–24]; and, 6) support vector clustering [25–27].

In the recent literature, support vector clustering has gained a lot of attention [26–31]. However, it is expensive in processing time and sometimes fails to find meaningful clusters. In general, clustering methods based on Bayes classifier and maximum likelihood are still the preferred choice compared to support vector clustering for many applications. There are various approaches to implement these clustering methods.

In this paper, we focus on maximum likelihood estimate. There are three ways to implement the maximum likelihood method. 1) Analytic way: likelihood functions are differentiated and equated to zero and the equations are solved to find extrema. The second derivative is then taken to ensure if maxima has reached rather than minima. 2) Grid search: an exhaustive search over a region is conducted to find the parameters that produce largest likelihood. 3) Numerical analysis: an initial value of parameter is used in a hill climbing algorithm or gradient ascent algorithm (e.g. Newton-Rapson, BHHH, DFP) to find the maxima. Maximum likelihood is also estimated via EM algorithm [5, 22, 32–39].

In general, it is impossible to use an analytic approach to find maximum likelihood estimates as the parametric form of data is unknown. Grid search is only possible when the dimensionality of the data is very small. Most of the time, maximum likelihood is computed by a hill climbing algorithm or by the EM algorithm. The potential problem with gradient algorithms is that when likelihood is not differentiable then it is not possible to find gradient to convergence. Considering this, in this paper, we propose a stepwise iterative maximum likelihood (SIML) procedure which does not require derivatives of likelihood functions. It can find all unknown parameters without solving first derivative and second derivatives of likelihood. The experimental results also show promising when compared to many state-of-the-art clustering methods.

## Methods

### Description of Maximum Likelihood Clustering

Here, we briefly discuss maximum likelihood method for clustering [5]. Assume a *d* -dimensional sample set *χ* = {**x**_1_, **x**_2_, …, **x**_n_} having *n* unlabelled samples, and, *c* is the number of clusters. Let *Ω* = {*ω*_*j*_} (for *j* = 1, 2, …, *c*) be the state of the nature or class label for *j* th cluster *χ*_*j*_. Suppose **θ** = {**θ**_*j*_} (for *j* = 1 … *c*) is any unknown parameter (having mean **μ** and covariance *Σ*). Then the mixture density is given by

1 $$ p\left(\mathbf{x}\Big|\boldsymbol{\uptheta} \right)={\displaystyle {\sum}_{j=1}^cp\left(\mathbf{x}\Big|{\omega}_j,{\boldsymbol{\uptheta}}_j\right)P\left({\omega}_j\right)} $$

where *p*(**x**|*ω*_*j*_, **θ**_*j*_) is the conditional density, and *P*(*ω*_*j*_) is the a priori probability. The log likelihood can be represented by joint density



Suppose that the joint density  is differentiable with respect to **θ** then from Eqs. 1 and 2

3 $$ {\mathit{\nabla}}_{{\boldsymbol{\uptheta}}_i}L={\displaystyle {\sum}_{k=1}^n\frac{1}{p\left({\mathbf{x}}_k\Big|\boldsymbol{\uptheta} \right)}{\mathit{\nabla}}_{{\boldsymbol{\uptheta}}_{\boldsymbol{i}}}\left[{\displaystyle {\sum}_{j=1}^cp\left({\mathbf{x}}_k\Big|{\omega}_j,{\boldsymbol{\uptheta}}_j\right)P\left({\omega}_j\right)}\right]} $$

where ${\nabla}_{{\boldsymbol{\uptheta}}_i}L$ is the gradient of *L* with respect to **θ**_*i*_ . If **θ**_*i*_ and **θ**_*j*_ are independent and suppose a posteriori probability is given as

4 $$ P\left({\omega}_i\Big|{\mathbf{x}}_k,\boldsymbol{\uptheta} \right)=\frac{p\left({\mathbf{x}}_k\Big|{\omega}_i,{\boldsymbol{\uptheta}}_i\right)P\left({\omega}_i\right)}{p\left({\mathbf{x}}_k\Big|\boldsymbol{\uptheta} \right)} $$

then from Eqs. 3 and 4, we have

5 $$ {\mathit{\nabla}}_{{\boldsymbol{\uptheta}}_i}L={\displaystyle {\sum}_{k=1}^nP\left({\omega}_i\Big|{\mathbf{x}}_k,\boldsymbol{\uptheta} \right){\mathit{\nabla}}_{{\boldsymbol{\uptheta}}_i} \log p\left({\mathbf{x}}_k\Big|{\omega}_i,{\boldsymbol{\uptheta}}_i\right)} $$

The gradient of likelihood (Eq. 5) can be equated to zero (${\mathit{\nabla}}_{{\boldsymbol{\uptheta}}_i}L=0$) to obtain maximum likelihood estimate ${\widehat{\boldsymbol{\uptheta}}}_i$. The solution can be therefore obtained by

6 $$ P\left({\omega}_i\right)=\frac{1}{n}{\displaystyle {\sum}_{k=1}^nP\left({\omega}_i\Big|{\mathbf{x}}_k,\widehat{\boldsymbol{\uptheta}}\right)} $$

7 $$ {\displaystyle {\sum}_{k=1}^nP\left({\omega}_i\Big|{\mathbf{x}}_k,\widehat{\boldsymbol{\uptheta}}\right){\nabla}_{{\boldsymbol{\uptheta}}_i} \log p\left({\mathbf{x}}_k\Big|{\omega}_i,{\widehat{\boldsymbol{\uptheta}}}_i\right)=0} $$

8 $$ P\left({\omega}_i\Big|{\mathbf{x}}_k,\widehat{\boldsymbol{\uptheta}}\right)=\frac{p\left({\mathbf{x}}_k\Big|{\omega}_i,{\widehat{\boldsymbol{\uptheta}}}_i\right)P\left({\omega}_i\right)}{{\displaystyle {\sum}_{j=1}^cp\left({\mathbf{x}}_k\Big|{\omega}_j,{\widehat{\boldsymbol{\uptheta}}}_j\right)P\left({\omega}_j\right)}} $$

In the above equations, **θ** is replaced by unknown mean and covariance parameters for normal distribution case, to yield maximum likelihood estimates. In the literature, parameter **θ** is iteratively updated to reach the final value $\widehat{\boldsymbol{\uptheta}}$ using hill climbing algorithms such as the Newton-Raphson method. In general, the computation of first and second derivatives of likelihood is required to find the solution. If the likelihood is differentiable and the a priori probability is non-zero, then convergence can be obtained. However, there is always a possibility of being trapped in a local optima.

### Stepwise iterative maximum likelihood method

**Fig. 1:**
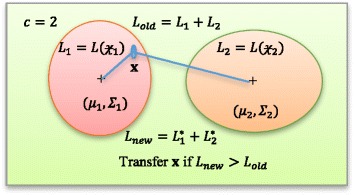
An illustration of stepwise iterative maximum likelihood method using a ***c*** = 2 cluster case. In this illustration, two clusters  and  are given with likelihood functions L_1_ and L_2_, respectively. The center of clusters are depicted by *μ*_1_ and *μ*_2_ (shown as ‘+’ inside two clusters). Initial total likelihood is L_old_ which is the sum of two likelihood functions (L_1_ + L_2_). A sample $\mathrm{x}\in$  is checked for grouping. It is advantageous to shift sample $\mathrm{x}$ to cluster  only if the new likelihood (L_new_ = *L*_1_^*^ + *L*_2_^*^) is higher than the old likelihood; i.e., *L*_*new*_ > *L*_*old*_

In this section, we describe our proposed method. This method seeks the most optimal partitions in an iterative way. We begin with an initial partition of data and shift a sample from one partition to another partition, and test if such a shift improves the overall log-likelihood. A simple illustration of SIML is given in Fig. 1.

If we define class-based log-likelihood of two clusters *χ*_*i*_ and *χ*_*j*_ as

9 $$ {L}_i={\displaystyle {\sum}_{\mathbf{x}\in {\chi}_i} \log \left[p\left(\mathbf{x}\Big|{\omega}_i,{\boldsymbol{\uptheta}}_i\right)P\left({\omega}_i\right)\right]} $$

and

10 $$ {L}_j={\displaystyle {\sum}_{\mathbf{x}\in {\chi}_j} \log \left[p\left(\mathbf{x}\Big|{\omega}_j,{\boldsymbol{\uptheta}}_j\right)P\left({\omega}_j\right)\right]}, $$

then we would be interested in knowing how the class-based log-likelihood functions (referred as log-likelihood function hereafter) change if a sample is shifted from *χ*_*i*_ to *χ*_*j*_. In order to know this, let us define mean and covariance of *χ*_*i*_ and *χ*_*j*_ as **μ**_*i*_ and **μ**_*j*_, and, as *Σ*_*i*_ and *Σ*_*j*_, respectively. The following equations describe mean and covariance:

11 $$ {\boldsymbol{\upmu}}_i=\frac{1}{n_i}{\displaystyle {\sum}_{\mathbf{x}\in {\chi}_i}\mathbf{x}} $$

12 $$ {\boldsymbol{\upmu}}_j=\frac{1}{n_j}{\displaystyle {\sum}_{\mathbf{x}\in {\chi}_j}\mathbf{x}} $$

13 $$ {\varSigma}_i=\frac{1}{n_i}{\displaystyle {\sum}_{\mathbf{x}\in {\chi}_i}\left(\mathbf{x}-{\boldsymbol{\upmu}}_i\right){\left(\mathbf{x}-{\boldsymbol{\upmu}}_i\right)}^{\mathrm{T}}} $$

and

14 $$ {\varSigma}_j=\frac{1}{n_j}{\displaystyle {\sum}_{\mathbf{x}\in {\chi}_j}\left(\mathbf{x}-{\boldsymbol{\upmu}}_j\right){\left(\mathbf{x}-{\boldsymbol{\upmu}}_j\right)}^{\mathrm{T}}} $$

where *n*_*i*_ and *n*_*j*_ are number of samples in *χ*_*i*_ and *χ*_*j*_, respectively. If the component density is normal and let *P*(*ω*_*i*_) = *n*_*i*_/*n* (where *n* is the total number of samples) then Eqs. 9 and 10 can be written as

$$ {L}_i={\displaystyle {\sum}_{\mathbf{x}\in {\chi}_i} \log \left[\frac{1}{{\left(2\pi \right)}^{d/2}{\left|{\varSigma}_i\right|}^{1/2}} \exp \left[-\frac{1}{2}{\left(\mathbf{x}-{\boldsymbol{\upmu}}_i\right)}^{\mathrm{T}}{\varSigma}_i^{-1}\left(\mathbf{x}-{\boldsymbol{\upmu}}_i\right)\right]\right]}+{n}_i \log P\left({\omega}_i\right)\mathrm{or}=-\frac{1}{2}tr\left[{\varSigma}_i^{-1}{\displaystyle {\sum}_{\mathbf{x}\in {\chi}_i}\left(\mathbf{x}-{\boldsymbol{\upmu}}_i\right){\left(\mathbf{x}-{\boldsymbol{\upmu}}_i\right)}^{\mathrm{T}}}\right]-\frac{n_id}{2} \log 2\pi -\frac{n_i}{2} \log \left|{\varSigma}_i\right|+{n}_i \log \frac{n_i}{n} $$

where *tr*() is a trace function. Since $tr\left[{\varSigma}_i^{-1}{\displaystyle {\sum}_{\mathbf{x}\in {\chi}_i}\left(\mathbf{x}-{\boldsymbol{\upmu}}_i\right){\left(\mathbf{x}-{\boldsymbol{\upmu}}_i\right)}^{\mathrm{T}}}\right]=tr\left({n}_i{I}_{d\times d}\right)={n}_id$ we can write *L*_*i*_ as

15 $$ {L}_i=-\frac{1}{2}{n}_id-\frac{n_id}{2} \log 2\pi -\frac{n_i}{2} \log \left|{\varSigma}_i\right|+{n}_i \log \frac{n_i}{n} $$

Similarly, we can write *L*_*j*_ as

16 $$ {L}_j=-\frac{1}{2}{n}_jd-\frac{n_jd}{2} \log 2\pi -\frac{n_j}{2} \log \left|{\varSigma}_j\right|+{n}_j \log \frac{n_j}{n}, $$

and the total log-likelihood for *c* clusters can be written as

17 $$ {L}_{tot}={\displaystyle {\sum}_{k=1}^c{L}_k} $$

where *L*_*k*_ is from Eq. 15 or 16.

If a sample $\widehat{\mathbf{x}}\in {\chi}_i$ is shifted to *χ*_*j*_, then the mean and covariance will change as follows (from Eqs. 11, 12, 13 and 14):

18 $$ {\boldsymbol{\upmu}}_j^{*}={\boldsymbol{\upmu}}_j+\frac{\widehat{\mathbf{x}}-{\boldsymbol{\upmu}}_j}{n_j+1} $$

19 $$ {\boldsymbol{\upmu}}_i^{*}={\boldsymbol{\upmu}}_i-\frac{\widehat{\mathbf{x}}-{\boldsymbol{\upmu}}_i}{n_i-1} $$

20 $$ {\varSigma}_j^{*}=\frac{n_j}{n_j+1}{\varSigma}_j+\frac{n_j}{{\left({n}_j+1\right)}^2}\left(\widehat{\mathbf{x}}-{\boldsymbol{\upmu}}_j\right){\left(\widehat{\mathbf{x}}-{\boldsymbol{\upmu}}_j\right)}^{\mathrm{T}} $$

21 $$ {\varSigma}_i^{*}=\frac{n_i}{n_i-1}{\varSigma}_i-\frac{n_i}{{\left({n}_i-1\right)}^2}\left(\widehat{\mathbf{x}}-{\boldsymbol{\upmu}}_i\right){\left(\widehat{\mathbf{x}}-{\boldsymbol{\upmu}}_i\right)}^{\mathrm{T}} $$

where **μ**_i_^*^, **μ**_j_^*^, Σ_i_^*^ and *Σ*_j_^*^ are means and covariances after the shift.

In order to find the change in log-likelihood functions *L*_*i*_ and *L*_*j*_, we first introduce the following Lemma.

**Lemma 1** If a sample $\widehat{\mathbf{x}}\in {\chi}_i$ is shifted to cluster *χ*_*j*_ and the changed covariance of *χ*_*j*_ is defined as ${\varSigma}_j^{*}=\frac{n_j}{n_j+1}{\varSigma}_j+\frac{n_j}{{\left({n}_j+1\right)}^2}\left(\widehat{\mathbf{x}}-{\boldsymbol{\upmu}}_j\right){\left(\widehat{\mathbf{x}}-{\boldsymbol{\upmu}}_j\right)}^{\mathrm{T}}$ then the determinant of *Σ*_j_^*^ can be given as $\left|{\varSigma}_j^{*}\right|={\left(\frac{n_j}{n_j+1}\right)}^d\left|{\varSigma}_j\right|\left(1+\frac{1}{n_j+1}{\left(\widehat{\mathbf{x}}-{\boldsymbol{\upmu}}_j\right)}^{\mathrm{T}}{\varSigma}_j^{-1}\left(\widehat{\mathbf{x}}-{\boldsymbol{\upmu}}_j\right)\right)$.

*Proof* By taking determinant of *Σ*_j_^*^, we get

L1 $$ \left|{\varSigma}_j^{*}\right|=\left|\frac{n_j}{n_j+1}{\varSigma}_j+\frac{n_j}{{\left({n}_j+1\right)}^2}\left(\widehat{\mathbf{x}}-{\boldsymbol{\upmu}}_j\right){\left(\widehat{\mathbf{x}}-{\boldsymbol{\upmu}}_j\right)}^{\mathrm{T}}\right| $$

since for *m* × *m* square matrices |*AB*| = |*A*||*B*| and for a scalar *c*, |*cA*| = *c*^*m*^|*A*|. We can write Eq. L1 as

L2 $$ \left|{\varSigma}_j^{*}\right|={\left(\frac{n_j}{n_j+1}\right)}^d\left|{\varSigma}_j\right|\left|{I}_{d\times d}+\frac{1}{n_j+1\ }\left(\widehat{\mathbf{x}}-{\boldsymbol{\upmu}}_j\right){\left(\widehat{\mathbf{x}}-{\boldsymbol{\upmu}}_j\right)}^{\mathrm{T}}{\varSigma}_j^{-1}\right| $$

From Sylvester’s determinant theorem, rectangular matrices *A* ∈ ℝ^*m* × *n*^ and *B* ∈ ℝ^*n* × *m*^ in |*I*_*m* × *m*_ + *AB*| is equal to |*I*_*n* × *n*_ + *BA*|. Therefore, we can write

L3 $$ \left|{I}_{d\times d}+\frac{1}{n_j+1\ }\left(\widehat{\mathbf{x}}-{\boldsymbol{\upmu}}_j\right){\left(\widehat{\mathbf{x}}-{\boldsymbol{\upmu}}_j\right)}^{\mathrm{T}}{\varSigma}_j^{-1}\right|=1+\frac{1}{n_j+1\ }{\left(\widehat{\mathbf{x}}-{\boldsymbol{\upmu}}_j\right)}^{\mathrm{T}}{\varSigma}_j^{-1}\left(\widehat{\mathbf{x}}-{\boldsymbol{\upmu}}_j\right) $$

since |*c*| = *c*.

Substituting right hand side of Eq. L3 in Eq. L2 proves the Lemma.

As similar to Lemma 1, the determinant of the change in covariance of *χ*_*i*_ can be written as

22 $$ \left|{\varSigma}_i^{*}\right|={\left(\frac{n_i}{n_i-1}\right)}^d\left|{\varSigma}_i\right|\left(1-\frac{1}{n_i-1}{\left(\widehat{\mathbf{x}}-{\boldsymbol{\upmu}}_i\right)}^{\mathrm{T}}{\varSigma}_i^{-1}\left(\widehat{\mathbf{x}}-{\boldsymbol{\upmu}}_i\right)\right) $$

We can now observe the change in *L*_*j*_ (Eq. 16) due to the shift of a sample $\widehat{\mathbf{x}}$ from *χ*_*i*_ to *χ*_*j*_ as

23 $$ {L}_j^{*}=-\frac{1}{2}\left({n}_j+1\right)d-\frac{\left({n}_j+1\right)d}{2} \log 2\pi -\frac{n_j+1}{2} \log \left|{\varSigma}_j^{*}\right|+\left({n}_j+1\right) \log \frac{n_j+1}{n} $$

From Lemma 1 and Eq. 16, we can rewrite Eq. 23 after doing algebraic manipulation as

24 $$ {L}_j^{*}={L}_j+\left(\varDelta {L}_j+C\right) $$

where *ΔL*_*j*_ is given by

25 $$ \varDelta {L}_j=-\frac{1}{2} \log \left|{\varSigma}_j\right|-\frac{n_j+1}{2} \log \left(1+\frac{1}{n_j+1}{\left(\widehat{\mathbf{x}}-{\boldsymbol{\upmu}}_j\right)}^{\mathrm{T}}{\varSigma}_j^{-1}\left(\widehat{\mathbf{x}}-{\boldsymbol{\upmu}}_j\right)\right)+ \log \frac{n_j}{n}+\left({n}_j+1\right)\left(\frac{d}{2}+1\right) \log \frac{n_j+1}{n_j} $$

and constant *C* is given by

26 $$ C=-\frac{d}{2}-\frac{d}{2} \log 2\pi $$

In a similar manner, change in *L*_*i*_ can be obtained by using Eqs. 15 and 22 as

27 $$ {L}_i^{*}={L}_i-\left(\varDelta {L}_i+C\right) $$

where *ΔL*_*i*_ is given by

28 $$ \varDelta {L}_i=-\frac{1}{2} \log \left|{\varSigma}_i\right|+\frac{n_i-1}{2} \log \left(1-\frac{1}{n_i-1}{\left(\widehat{\mathbf{x}}-{\boldsymbol{\upmu}}_i\right)}^{\mathrm{T}}{\varSigma}_i^{-1}\left(\widehat{\mathbf{x}}-{\boldsymbol{\upmu}}_i\right)\right)+ \log \frac{n_i}{n}-\left({n}_i-1\right)\left(\frac{d}{2}+1\right) \log \frac{n_i-1}{n_i} $$

and *C* is same as of Eq. 26.

By adding Eqs. 24 and 27, we can get the change in total log-likelihood (*L*_tot_^*^) since there is no change in any other clusters apart from *χ*_*i*_ to *χ*_*j*_; i.e., from Eqs. 17, 24 and 27 we have

29 $$ {L}_{tot}^{*}={L}_{tot}+\varDelta {L}_{tot} $$

where *ΔL*_*tot*_ = *ΔL*_*j*_ − *ΔL*_*i*_. Therefore, the shift of a sample $\widehat{\mathbf{x}}$ is advantageous if *ΔL*_*tot*_ > 0. This will give the following algorithm (Table 1):

**Table 1:** Stepwise iterative maximum likelihood method procedure

1. *Initialization*: select initial partitions with means *μ*_1_, *μ*_2_, …, *μ*_*c*_ and covariance matrices *Σ*_1_, *Σ*_2_, …, *Σ*_*c*_	
2. *Loop*: Select a sample $\widehat{\mathbf{x}}\in {\chi}_i$.	
3. If *n*_*i*_ > 1 then compute	
4. $$ {\delta}_j=\left\{\begin{array}{c}\hfill \varDelta {L}_j,\kern0.75em j\ne i\hfill \\ {}\hfill \varDelta {L}_i,\kern0.75em j=i\hfill \end{array}\right. $$	
5. Transfer $\widehat{\mathbf{x}}$ to *χ*_*k*_ if *δ*_*k*_ = max *δ*_*j*_ for all *j*.	
6. Update *L*_*tot*_, *μ*_*i*_, *μ*_*k*_, *Σ*_*i*_ and *Σ*_*k*_.	
7. If *L*_*tot*_ doesn’t change in *n* attempts then stop otherwise go to Loop.	

The following sections discuss the characteristic of the SIML method.

### Initial settings of the procedure

Similar to any other iterative based optimization technique, this technique also depends on the initial settings. Therefore, it is important to put consideration into the initial settings. In this paper, we implemented three ways of initializing the partitions: 1) random initialization, 2) *k*-means based initialization, and 3) initialization based on the solution of *c* − 1 partitions and the mean. These schemes are described as follows:

1. Random initialization: In this scheme, we create *c* random means around the center of the data. This technique works well when the number of clusters is small. If *c* is very large then it can miss clusters.
2. *K*-means initialization: In this scheme, the data is first partitioned into *c* clusters by using the k-means algorithm. The solution of *k*-means is used as an initial setting for the SIML method. This method works well even if the number of clusters is large. Most of the time this initialization technique provides good results. However, since the k-means algorithm does not track the data based on covariance information, it has limitations.
3. Initialization based on the solution of *c* − 1 clusters: The initialization of *c* clusters is done by using the solution of *c* − 1 clusters, which would give *c* − 1 locations. The *c* th location is the mean of the overall data itself. If only two clusters are required to find, then 2 locations around center of the data is used since the solution of 1-cluster is the center of the data itself.

In this paper, we used all the three schemes for initialization and in general schemes 2 and 3 provide satisfactory results for most of the data conformations.

### Numerical stability

Due to numerical difficulties the convergences of an iterative algorithm can be missed (e.g. convergence problem for EM algorithm is discussed in [40]). The problem of numeral difficulties is of particular issue when data dimensionality is high. In this situation, iterative algorithms sometimes do not converge properly. This problem usually appears due to the small numerical values of the covariance matrix. If the eigenvalues of a covariance matrix *Σ* are small, then its determinant can give a value close to zero due to the fixed point architecture of the hardware. However, this problem can be easily overcome by first conducting eigenvalue decomposition of *Σ* and using the summation of the logarithm of eigenvalues. It is described as follows:

The eigenvalue decomposition of *Σ* ∈ ℝ^*d* × *d*^ will give EDE^T^ where E ∈ ℝ^d × d^ is the eigenvector matrix and D ∈ ℝ^*d* × *d*^ is the diagonal matrix of eigenvalues. The determinant of *Σ* will be

$$ \left|\varSigma \right|=\left|{\mathrm{EDE}}^{\mathrm{T}}\right|=\left|\mathrm{D}\right|={\displaystyle \prod_{k=1}^d}{\lambda}_k $$

where *λ*_*k*_ is the *k* th eigenvalue of *Σ*. If the values of *λ* are small then |*Σ*| = 0. This problem can be overcome by simply taking logarithm as

$$ \log \left|\varSigma \right|={\displaystyle \sum_{k=1}^d} \log {\lambda}_k $$

In a similar way, the inverse of *Σ* can cause problems in the term of Eq. 28; i.e., the computation of the term $\log \left(1-\frac{1}{n_i-1}P\right)$ (where $P={\left(\widehat{\mathbf{x}}-{\boldsymbol{\upmu}}_i\right)}^{\mathrm{T}}{\varSigma}_i^{-1}\left(\widehat{\mathbf{x}}-{\boldsymbol{\upmu}}_i\right)$) when the size of the covariance matrix is large. In order to make this numerically stable a small quantity ϵ > 0 can be included as follows:

$$ \log \left(1-\frac{1}{n_i-1+\epsilon }P\right) $$

This will ensure that $1-\frac{P}{n_i+1+\epsilon }>0$.

### Small sample size case

When the dimensionality *d* is much greater than the number of samples *n* (*d* ≫ *n*) then small sample size problem appears [41–44]. Let a sample set *χ* = {**x**_1_, **x**_2_, …, **x**_*n*_} be drawn independently and let the mean and covariance of *χ* be denoted by **μ** and *Σ*, respectively. In the normal density we have a term *P* = (**x** − **μ**)^T^*Σ*^− 1^(**x** − **μ**) to compute which cannot be solved due to singular covariance matrix as its inverse does not exist. A simple extension could be to use the pseudo-inverse of *Σ* (denoted here as *Σ*^+^). However, this doesn’t solve the problem. If samples **x** are from *χ* then *P*^+^ = (**x** − **μ**)^T^*Σ*^+^(**x** − **μ**) will always be equal to the rank of *Σ* or basically *n* − 1 (for *d* ≫ *n*). This means that all the samples in a particular cluster would have the same probability and it would not be possible to justify classification of samples based on probability. A second way would be to regularize *Σ*, however, computing optimal regularization parameter could be a challenging task. One way could be to apply principal component analysis (PCA) procedure on a *d* -dimensional sample set *χ* ∈ ℝ^*d*^ to transform it to a parsimonious sample set *Y* ∈ ℝ^*h*^ where *h* < min(*d*, *n*) *h* < min(*d*, *n*). Thereafter, the clustering procedure can be performed.

### Determination of the number of clusters

It is potentially important to estimate the number of clusters *c* present in the sample set. Since this information is usually not provided, it is important to obtain the value of *c* with whatever information we have at hand. Basically, the only information we have is the sample itself. In the maximum likelihood procedure we compute likelihood from sample set. Therefore, this information can be utilized to estimate the number of clusters. In order to evaluate *c*, we can run the SIML algorithm for a range of clusters e.g. 1 ≤ *c* ≤ *K* to see at what point the likelihood function stabilizes or reaches maximum. In this paper, we investigated two ways to compute *c*. In the first way, we compute the maximum log-likelihood *MaxL*_*tot*_(*c*) achieved for all values of *c* ∈ [1, *K*]. At a particular value of *c* the *MaxL*_*tot*_ reaches maximum and does not change much. This would be the estimated value of *c*. In the second way, we compute the difference between the maximum log-likelihood *MaxL*_*tot*_ achieved and the first value of *L*_*tot*_ after SIML procedure (excluding the initial *L*_*tot*_ value computed from initial settings as this value is based on the first random guess). Therefore, for a particular number of cluster *c*, we will get this difference likelihood and we denote it as *DelL*_*tot*_ which is equal to *MaxL*_*tot*_ − *L*_*tot*_(1) or max(*L*_*tot*_) − *L*_*tot*_(1), where *L*_*tot*_(*r*) defines the value of *L*_*tot*_ at an iteration *r*. The curve of *DelL*_*tot*_ as a function of *c* would give a peak at some value of *c* which would be its best value. In most of the data conformations, *MaxL*_*tot*_ gives reliable results. Nonetheless, both the graphs of *MaxL*_*tot*_ and *DelL*_*tot*_ (as a function of *c*) are illustrated in the experimental section of the paper.

## Results

In order to evaluate the algorithm, we carried out experiments on normal Gaussian data as well as on biological data. We divide this section into 5 subsections. In subsection 1, we illustrate the performance of various methods using three cluster case. Subsection 2 indulges on maximum likelihood plots as a function of number of clusters. In subsection 3, we discuss the processing time of the algorithm. In subsection 4, we discuss the performance in terms of clustering accuracy and rand score of various methods; and, in subsection 5 (parts I and II), we discuss SIML on biological data.

### An illustration using three clusters

**Fig. 2:**
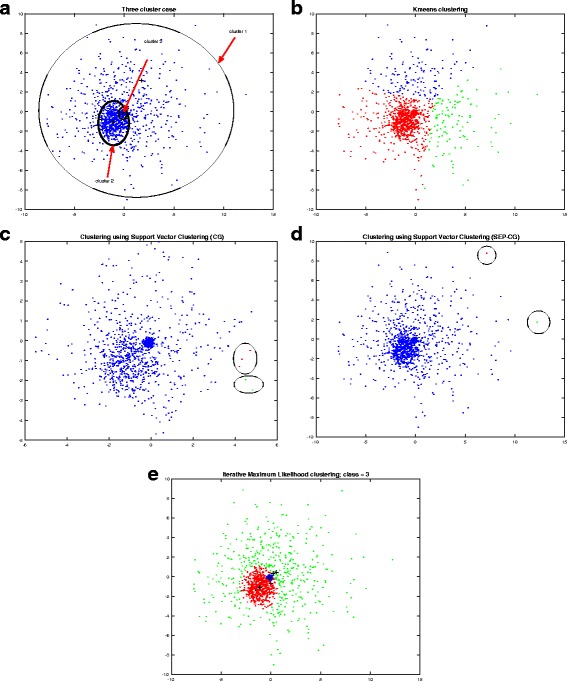
An illustration using 3 clusters: **a** Three cluster data where *n* = 1500 and *d* = 2; **b** k-means clustering, different colors show different clusters; **c** Support vector clustering (CG method); **d** Support vector clustering (SEP method); **e** Stepwise iterative maximum likelihood (SIML) method

Since data distribution of GWAS can appear as approximately Gaussian, we generated normal distribution data with 3 different mean and covariance for simulation purposes. Furthermore, if we consider GWAS data from a continent (e.g. Europe) as one cluster, from a country (e.g. Germany) as second cluster and from a city (e.g. Berlin) as a third cluster, then third cluster (Berlin data) will reside inside second cluster (Germany data), and second cluster (Germany data) will reside inside the first cluster (European data). Therefore, clusters will overlap with each other. To simulate this scenario, we generated a sample set with 1, 500 samples, 2 dimensions and 3 clusters as shown in Fig. 2a, and applied various methods on it. Cluster 1 is the least dense (or sparse) and Cluster 3 is the most dense. Cluster 1 has mean [0.1, 0.1] and variance 3 in each direction. Similarly, mean and variance of Cluster 2 and Cluster 3 were [−1, − 1] and 0.8, and, [−0.1, − 0.1] and 0.05, respectively. The clusters overlap each other and the goal is to track these clusters. It can be seen (from Fig. 2b) that *k*-means clustered the 3 clusters without considering the distribution information. The processing time to perform the k-means algorithm was 0.82 s. Support vector clustering (CG method) [25] was difficult to perform as it is not possible to provide number of class information. The parameters were tuned so that 3 clusters are outputed. The processing time by this method was 1183.1 s (excluding the tuning time). It can be observed from the Fig. 2c that this method was failed to track the clusters. Next, support vector clustering (using SEP method) [26] was performed. The default parameters gave 45 clusters. Therefore, as similar to the previous CG method, tuning of parameters was carried out to extract only 3 clusters. Processing time was 25.2 s excluding the tuning time. This method also misses the clusters (Fig. 2d). Then we performed the proposed SIML method. This method was able to track all the 3 clusters in 4.49 s per repetition (Fig. 2e). The likelihood plots are discussed in the following section.

### Likelihood plots

**Fig. 3:**
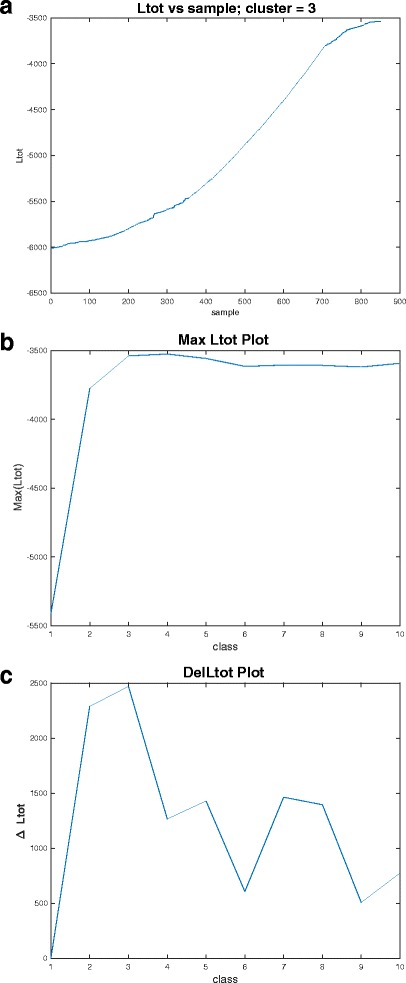
Likelihood plots **a** *L*_*tot*_ plot, **b** *MaxL*_*tot*_ plot and **c** *DelL*_*tot*_ plot

Here we discussed three plots: log-likelihood (*L*_*tot*_) versus sample (Fig. 3a), maximum log-likelihood (*MaxL*_*tot*_) as a function of number of clusters (Fig. 3b) and *DelL*_*tot*_ as a function of number of clusters (Fig. 3c).

Figure 3a depicts *L*_*tot*_ plot for 3 clusters. When a sample is moved from one cluster to another cluster the value of *L*_*tot*_ is updated. This is an increasing function and the maximum value of *L*_*tot*_ is defined as *MaxL*_*tot*_ in this paper.

Figure 3b depicts *MaxL*_*tot*_ plot. Since in general, the number of cluster *c* information is unknown, it is therefore crucial to estimate this value. In this paper we showed that by using *MaxL*_*tot*_ plot and *DelL*_*tot*_ plot, it is possible to estimate *c*. For this, one can provide a range of *c* values and the value for which *MaxL*_*tot*_ curve converges (reaches highest peak or does not change much) is the estimated *c*. We use the same data we generated in Fig. 2a and provide 10 values of *c* as 1 ≤ *c* ≤ 10. It can be seen from *MaxL*_*tot*_ plot that it converges or peaks at *c* = 3.

### Processing time

**Fig. 4:**
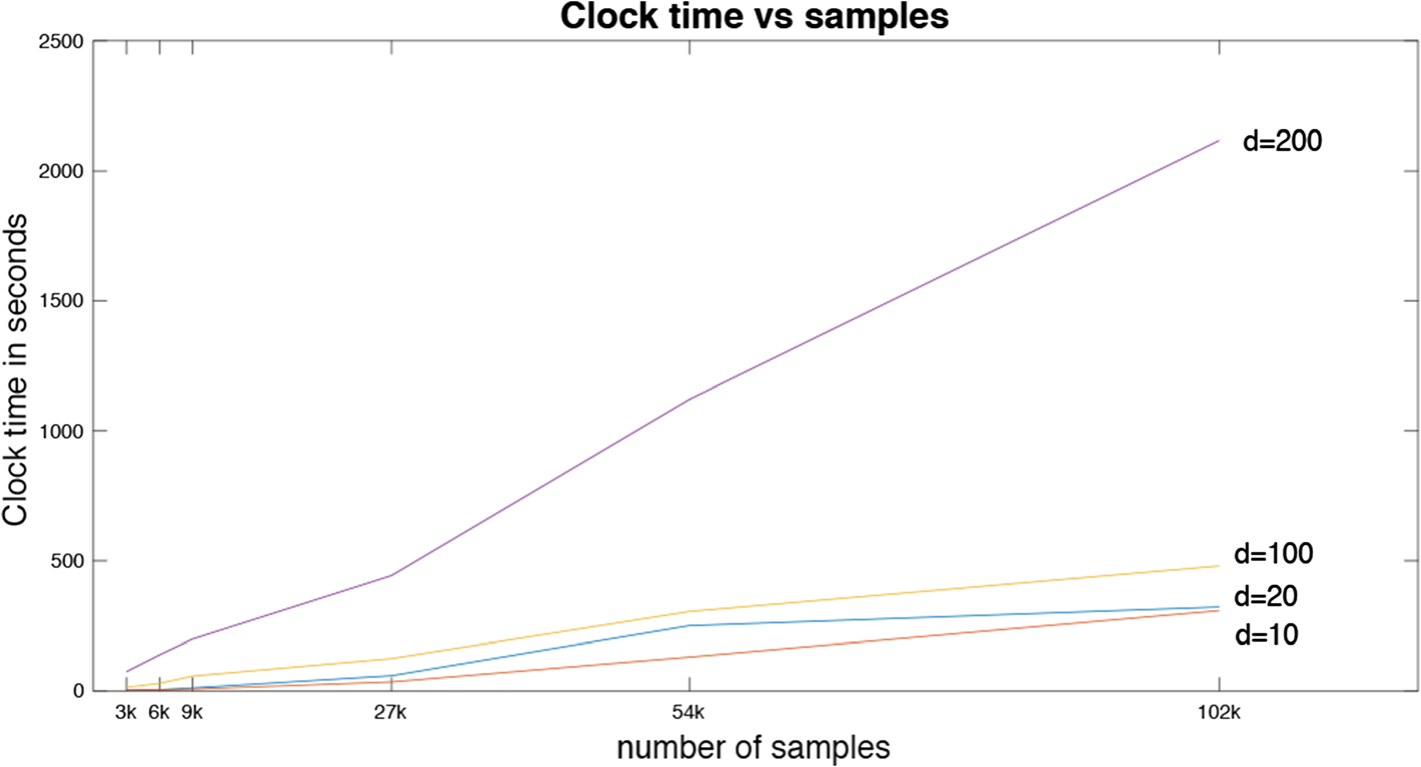
Processing time of SIML method for *n* = 3*k* − 102*k* and *d* = 10 − 200

Here we discuss the processing time of the SIML algorithm. In order to give a complete picture, we investigated the clock time in seconds for samples *n* = 3, 000, 9, 000, 27,000, 54,000 and 102,000 having 3 clusters. We use the same conformation of data as depicted in Fig. 2a, however, we increased the dimensionality to *d* = 10, 20, 100 and 200. Figure 4 shows the processing time of the algorithm when processed in Linux platform (Ubuntu 14.04 LTS, 64 bit) with 6 processors (Intel Xeon R CPU E5-1660 v2 @ 3.70 GHz) and with 128 GB memory for a repetition.

### Clustering on artificial data

**Fig. 5:**
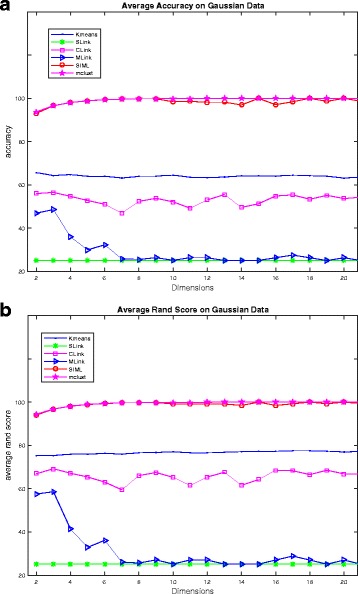
**a** Average clustering accuracy on Gaussian data. **b** Average rand score on Gaussian data

We performed clustering accuracy and rand score test on a set of artificial data. For artificial data, we generated *d* -dimensional, 4 cluster data such that cluster samples are overlapping to each other (in a similar way as shown in Fig. 2). There are in total 2000 samples (where each cluster having 500 samples). We computed cluster accuracy and rand score for various methods. For statistical stability, we generated data 20 times for a particular dimension *d* by changing random seed of the normal data. Thereby, we computed average clustering accuracy and average rand score over these 20 attempts for dimension *d*. We then varied dimension *d* = 2, 3, …, 20 and reported average clustering accuracy and average rand score in Fig. 5. For comparison, we used centroid-based technique like k-means, hierarchical-based technique like SLink [14], CLink [15] and MLink [16] and model-based technique (using EM algorithm) like mclust [39]. It can be observed from Fig. 5a that mclust and SIML methods perform quite well on Gaussian data. K-means algorithm also performs reasonably well on this data. MLink and SLink couldn’t perform well. For average rand score (Fig. 5b), CLink, *k*-means, SIML and mclust are exhibiting reasonable performance. However, mclust and SIML are superior reaching almost 100 rand score. Since mclust and SIML are derived from Gaussian model, their performance on Gaussian data are well compared to other techniques.

### Clustering on real data-I (publically available biological data)

In this section, we utilized various biological data and reported clustering accuracy and rand score. We employed several methods such as k-means, SLink, CLink, MLink and mclust for comparison. The description of biological data is given as follows:

SRBCT dataset [45]: the small round blue-cell tumor dataset consists of the expression of 2308 genes from 83 samples. This is a four class classification problem. The tumors are Burkitt lymphoma (BL), the Ewing family of tumors (EWS), neuroblastoma (NB) and rhabdomyosarcoma (RMS). The dataset consists of 11, 29, 18 and 25 samples of BL, EWS, NB and RMS respectively.

MLL leukemia [46]: This dataset has 3 classes, namely ALL, MLL and AML leukemia. The dataset contains 24 ALL, 20 MLL and 28 AML. The dimension of MLL dataset is 12,582.

ALL subtype dataset [47]: this dataset consists of the expression of 12,558 genes of subtypes of acute lymphoblastic leukemia. The dataset has seven classes namely BCR-ABL, E2A-PBX1, hyperdiploid >50 chromosomes ALL, MLL, T-ALL, TEL-AML1 and other (contains diagnostic samples that did not fit into any of the former six classes). Samples per class are 15, 27, 64, 20, 43, 79 and 79 respectively.

To vary the data dimensionality (number of features), we utilized Chi-squared feature selection method to rank the attributes. The dimensionality investigated was *d* = 2, 3, …, *n*_*m*_/2, where *n*_*m*_ is the cluster with minimum number of samples. We then performed cluster analysis (to evaluate clustering accuracy and rand score) on these datasets and compared SIML with the *k*-means, SLink, CLink, MLink and mclust methods. The results are reported in Tables 2 and 3 (for SRBCT dataset), Tables 4 and 5 (for MLL dataset), Tables 6 and 7 (ALL subtype dataset) and Table 8 (for estimation of number of clusters by SIML). Clustering accuracy is depicted in Tables 2, 3, 4, 5, 6 and rand score is shown in Tables 2, 3, 4, 5, 6 and 7. The methods achieving highest results are depicted in bold faces.

**Table 2:** Clustering accuracy on SRBCT dataset

Dim	K-means	SLINK	CLINK	MLINK	mclust	SIML
2	60.4	34.9	62.7	54.2	62.7	**63.9**
3	67.9	39.8	69.9	**71.1**	69.9	66.3
4	77.1	49.4	65.1	67.5	72.3	**81.9**
5	70.3	50.6	**72.3**	50.6	65.1	67.5
6	64.0	39.8	53.0	53.0	57.8	**69.9**

The methods achieving highest results are depicted in bold faces

**Table 3:** Rand score on SRBCT dataset

Dim	K-means	SLINK	CLINK	MLINK	mclust	SIML
2	69.5	32.9	69.9	60.0	62.7	**68.5**
3	77.2	32.0	76.6	**78.4**	69.9	75.3
4	80.5	51.3	71.4	74.8	72.3	**82.7**
5	78.3	53.1	**81.8**	53.1	65.1	75.0
6	72.4	35.8	56.5	56.5	57.8	**78.7**

The methods achieving highest results are depicted in bold faces

**Table 4:** Clustering accuracy on MLL dataset

Dim	K-means	SLINK	CLINK	MLINK	mclust	SIML
2	56.3	40.3	45.8	45.8	**80.6**	58.3
3	58.8	40.3	50.0	50.0	**68.1**	61.1
4	59.5	43.1	54.2	43.1	**95.8**	72.2
5	81.9	43.1	72.2	69.4	94.4	**95.8**
6	81.9	43.1	81.9	69.4	55.6	**95.8**
7	80.0	41.7	81.9	72.2	91.7	**94.4**
8	81.7	43.1	79.2	68.1	**90.3**	62.5
9	82.8	48.6	80.6	**84.7**	65.3	63.9
10	80.4	43.1	58.3	63.9	61.1	**91.7**

The methods achieving highest results are depicted in bold faces

**Table 5:** Rand score on MLL dataset

Dim	K-means	SLINK	CLINK	MLINK	mclust	SIML
2	63.6	35.0	41.1	41.1	**80.6**	72.3
3	67.5	35.0	45.7	45.7	68.1	**72.6**
4	64.0	36.3	47.2	36.3	**95.8**	77.5
5	80.4	36.3	75.2	70.2	94.4	**94.7**
6	80.4	36.3	80.4	70.2	55.6	**94.7**
7	79.6	35.3	80.4	75.7	91.7	**93.1**
8	80.6	36.3	78.4	67.7	**90.3**	69.9
9	81.2	41.1	79.3	**82.6**	65.3	71.6
10	80.3	36.3	66.1	73.2	61.1	**90.1**

The methods achieving highest results are depicted in bold faces

**Table 6:** Clustering accuracy on ALL subtype dataset

Dim	K-means	SLINK	CLINK	MLINK	mclust	SIML
2	**44.8**	32.1	42.8	36.1	34.3	44.0
3	53.3	25.1	45.3	46.2	34.9	**56.9**
4	57.4	25.1	51.7	49.9	33.3	**61.5**
5	60.4	26.0	42.8	34.6	44.7	**62.4**
6	58.9	25.4	38.8	41.0	45.3	**63.6**
7	**58.7**	24.2	47.4	36.1	49.5	56.3
8	54.5	25.7	42.8	34.8	41.9	**61.2**

The methods achieving highest results are depicted in bold faces

**Table 7:** Rand score on ALL subtype dataset

Dim	K-means	SLINK	CLINK	MLINK	mclust	SIML
2	**73.1**	37.1	68.2	49.9	34.3	71.8
3	**79.2**	20.5	73.5	62.7	34.9	77.6
4	**81.6**	20.4	78.3	72.4	33.3	81.2
5	79.6	22.0	69.0	47.3	44.7	**82.8**
6	79.9	21.6	69.5	67.5	45.3	**83.1**
7	79.9	21.0	75.2	40.6	49.5	**80.2**
8	77.8	21.7	70.3	60.6	74.9	**82.2**

The methods achieving highest results are depicted in bold faces

**Table 8:** The estimation of the number of clusters by SIML

Dim	SRBCT	MLL	ALL subtype
2	4	3	7
3	4	2	7
4	4	2	8
5	4	3	4,7
6	2,4	3	7,9
7		3	3,8
8		3	7
9		3	
10		6	

It can be observed from Tables 2 and 3 that SIML achieved the highest clustering accuracy and rand score in 3/5 cases, and MLink and CLink achieved the highest performance in 1/5 case each. For the MLL dataset (Tables 4 and 5), mclust achieved the highest clustering accuracy and rand score in 4/9 cases and 3/9 cases, respectively. SIML was able to achieve 4/9 times highest clustering accuracy and rand score. Apart from SIML and mclust, k-means was also able to get reasonable performance especially for higher dimensions. For ALL subtype dataset (Tables 6 and 7), k-means achieved the highest clustering accuracy in 2/7 cases and highest rand score in 3/7 cases. SIML reported the highest clustering accuracy and rand score in 5/7 cases and 4/7 cases, respectively. These results show that SIML can perform reasonably well for many datasets employed in this work. In Table 8, we provided the summary of the number of clusters estimated by SIML. The corresponding *MaxL*_*tot*_ plots are given in the Additional file 1. It can be seen from Table 8 that SIML estimates correctly the number of clusters most of the time.

### Clustering on real data-II (SNPs data)

In this section, we attempt to illustrate the use of SIML on real data case. In practical situation, there are two problems to address in a dataset: 1) how many clusters are present; and, 2) what are the locations of the clusters? [48–50]. Sometimes, it is also necessary to identify or remove some sub-population from the data in order to solve the issue of population stratification. The existence of population stratification unmatched between cases and controls can produce false positives and negatives in GWAS [51]. For this exercise, we utilize data from a collection of 7001 individuals from the BioBank Japan (BBJ) project and 45 Japanese HapMap (JPT) samples [51]. The total number of single nucleotide polymorphisms (SNPs) was 140,387, genotyped via the Perlegen platform. We also included 45 Han Chinese HapMap (CHB) samples and merged these data using PLINK v1.9 (https://www.cog-genomics.org/plink2) on 140,367 common SNPs. Prior to PCA, we performed filtering using similar criteria as of that used by Yamaguchi-Kabata et al. [51]. We removed SNPs with a call rate < 99 %, a MAF < 0.01, and a Hardy-Weinberg equilibrium (HWE) exact test *p*-value > 10^− 6^. Individuals with missing calls for > 5 % of SNPs were also removed. After filtering, 6998 BBJ, 44 JPT and 45 CHB samples sharing 117,758 SNPs remained. Consequently, the population consists of 6891 main land Japan (Hondo) samples, 45 CHB samples and 151 Okinawa samples referred as the Ryukyu (RYU) cluster. The Hondo samples can be further subdivided into 628 Kyushu, 908 Kinki, 358 Tokai-Hokoriku, 3975 Kanto-Koshinetsu, 466 Tohoku, 512 Hokkaido and 44 JPT samples. The aim here is to classify RYU and CHB from Hondo so that Hondo only data can be explored for further analysis. We first performed PCA on the filtered data using the R package SNPRelate [52] to reduce the data dimensionality and conducted analysis on 2 dimensional data. Linkage disequilibrium (LD) pruning with a threshold of 0.2 was used to define a representative set of 32,090 SNPs for PCA.

In summary, this two dimensional data contain three clusters: Hondo, RYU and CHB. Here we first computed true positives (and its corresponding accuracy) for Hondo, RYU and CHB clusters. This would provide us information regarding correctly labelled samples in each cluster. For this purpose, we executed all the methods to provide 3 clusters of the data. The true positives for various methods are depicted in Table 9.

**Table 9:** True positives for Hondo, RYU and CHB cluster on BBJ and HapMap data

	Hondo	RYU	CHB
Methods	(6891)	(151)	(45)
	71.4 %	85.5 %	100 %
K-means	4922	129	45
	99.9 %	0 %	100 %
SLINK	6886	0	45
	97.9 %	92.7 %	100 %
CLINK	6746	140	45
	95.8 %	92.1 %	100 %
MLINK	6603	139	45
	97.3 %	94.7 %	100 %
SIML	6707	143	45
	66.8 %	94.7 %	0 %
mclust	4602	143	0

From Table 9, we can see that *k*-means was able to cluster all CHB samples correctly and also attained high true positive for the RYU cluster. However, it displayed comparatively inferior performance for the Hondo cluster. SLink reported very high true positive for Hondo and CHB clusters. However, it completely missed the RYU cluster. CLink, MLink and SIML were able to label all 45 samples of CHB correctly. SIML achieved the highest true positive for RYU among these 3 methods and CLink was slightly better (97.9 %) than SIML (97.3 %) for the Hondo cluster. In this case, mclust did not perform well. Nonetheless, mclust gave a high true positive for RYU cluster. It should be noted here that this data is highly imbalanced. Out of 7087 samples, 6891 samples belong to the Hondo cluster (i.e., almost 97 %) leaving only 3 % of samples for the RYU and CHB clusters. This imbalance creates a problem in a way that majority of samples turn to be labelled under the larger cluster leaving the smaller clusters. Nonetheless, SIML has shown encouraging results.

**Fig. 6:**
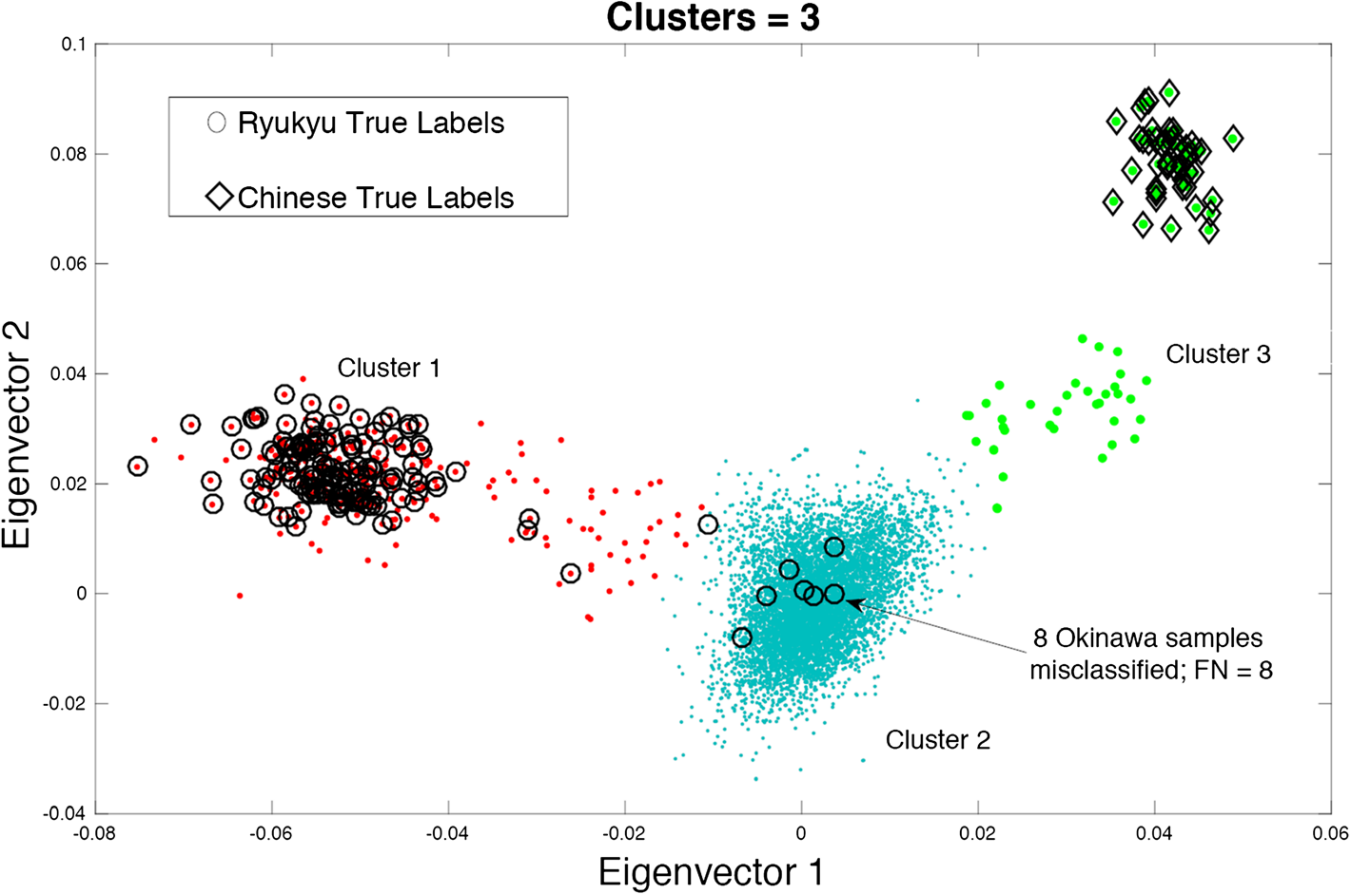
Clustering by SIML on 2-dimensional BBJ data

**Fig. 7:**
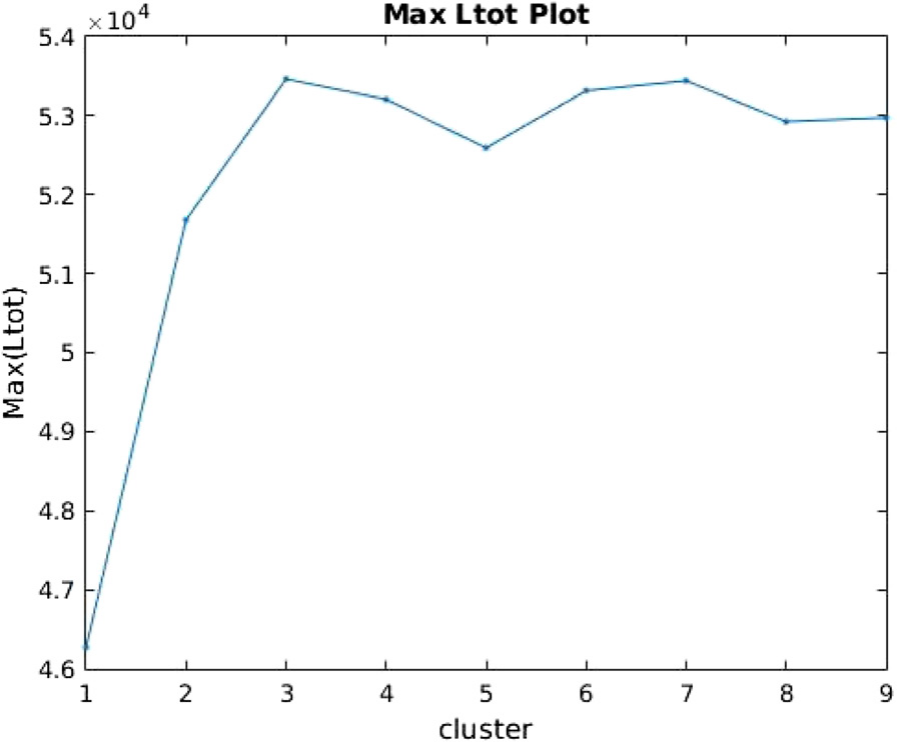
*MaxL*_*tot*_ Plot for 2-dimensional BBJ and HapMap data

In the next analysis, we did not provide the number of clusters information to study the characteristics of SIML method. The resulting clustering is illustrated in Fig. 6. For this case, the *MaxL*_*tot*_ plot gives peak at 3 clusters (Fig. 7) and therefore 3 clusters were used in this case. The true RYU and CHB labels are shown on the plot as circles and diamonds, respectively. Most of Hondo samples are in Cluster 2. There are around 6715 samples in Cluster 2 representing the Hondo region. Almost all CHB are clustered in Cluster 3 and most of RYU are clustered in Cluster 1. Around 8 RYU are clustered in Cluster 2 giving a false negative (FN) error of 8 samples (5.3 %) and no CHB sample is misclassified giving FN error of 0 samples (0 %). Cluster 1 and Cluster 3 can be classified easily and analysis can be conducted on Cluster 2 (Hondo) with very less FN error.

In summary, SIML successfully estimates the number of clusters as well as the locations. The SIML package was tested on Ubuntu 14.04 LTS OS (with 128 GB memory and Intel Xeon R CPU E5-1660 v2 @ 3.7 GHz x 6). The OS type is 64-bit. For Matlab we used ‘Statistics and Machine Learning Toolbox’.

## Conclusions

In this work, through considering conformations of many biological data, we developed a clustering algorithm based on maximum likelihood estimate. The proposed stepwise iterative maximum likelihood (SIML) method is different from other maximum likelihood methods as it does not require the computation of first and second derivative of likelihood functions. This avoids the necessity to have differentiable likelihood functions for convergence. The SIML method was tested on artificial and real data to evaluate its performance. We show that SIML was able to produce promising results over state-of-the-art methods. The SIML method was also able to estimate the number of clusters successfully. The Matlab package of SIML is available from our webpage.

## Additional file

Additional file 1: Estimation of number of clusters using SIML method. (DOCX 408 kb)
