# Supplementary material for: Stepwise iterative maximum likelihood clustering approach
Source: BMC Bioinformatics. 2016 Aug 24;17(1):319. doi: 10.1186/s12859-016-1184-5 (PMC4995791; doi:10.1186/s12859-016-1184-5)

**Supplementary 1**

**Estimation of number of clusters using SIML method**

In this supplementary document, SIML has been applied on three different datasets (SRBCT, MLL and ALL subtype) to investigate the most suited number of clusters. For SRBCT and MLL datasets we explored number of clusters in the range $[1,6]$ and for ALL subtype we explored in the range $[1,10]$. The $MaxL_{tot}$ plots for all these datasets over different dimensions are shown here under.

**SRBCT dataset**


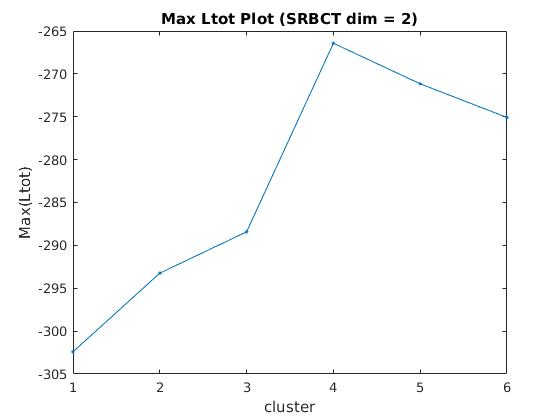


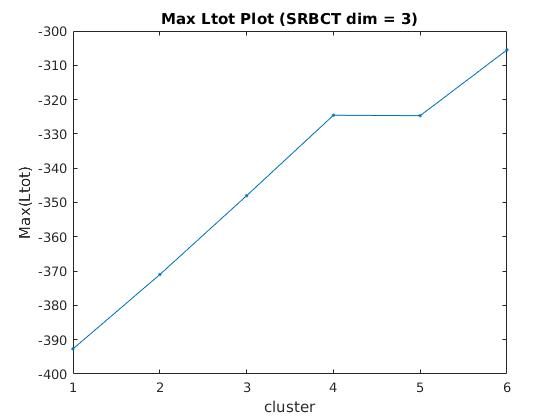


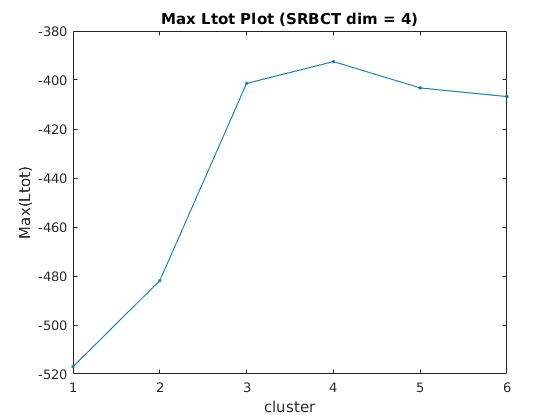


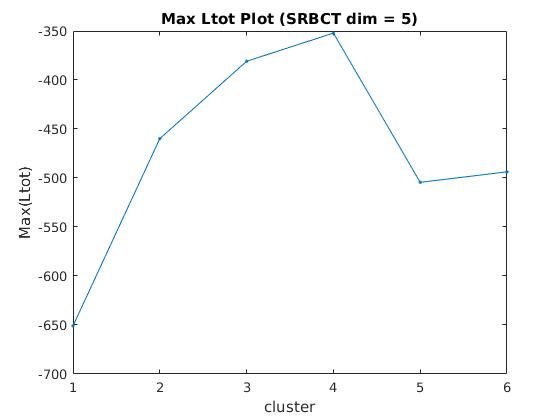


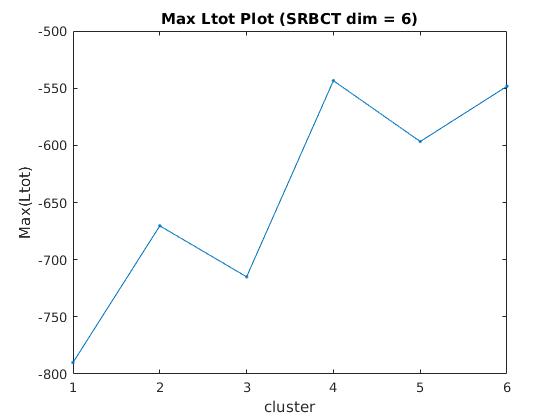


**MLL dataset**


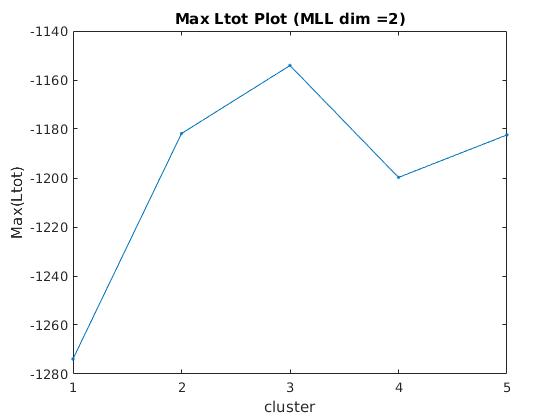


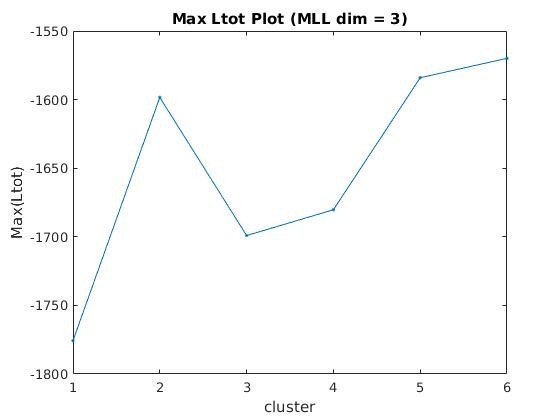


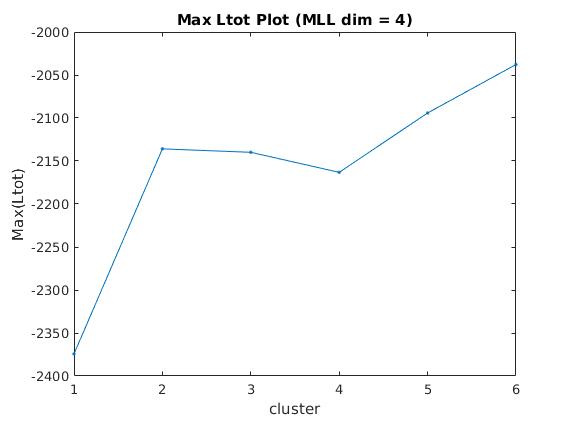


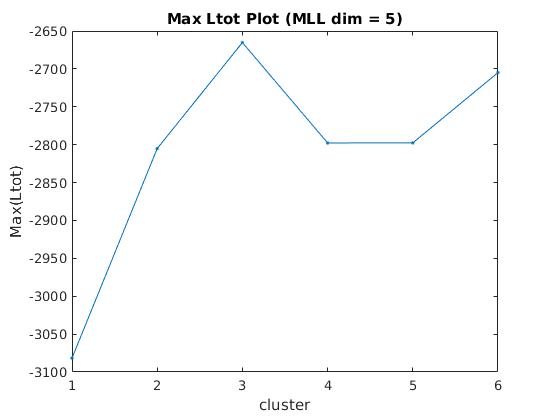


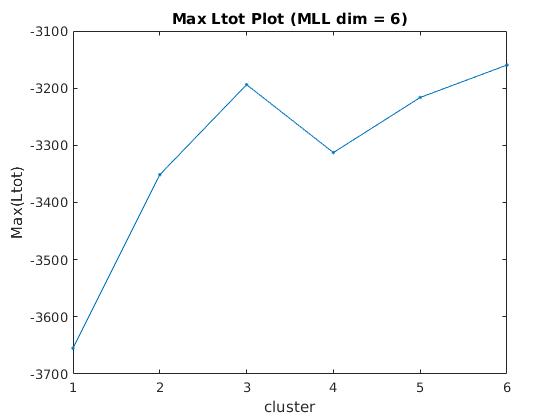


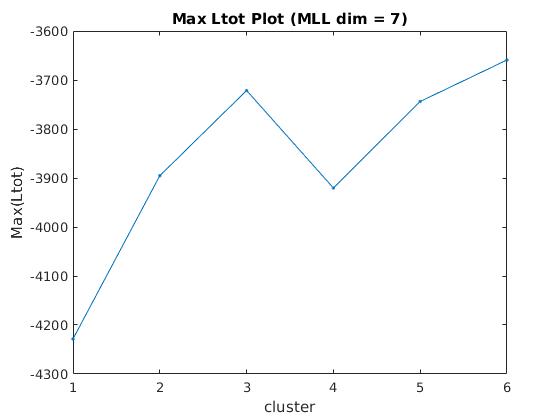


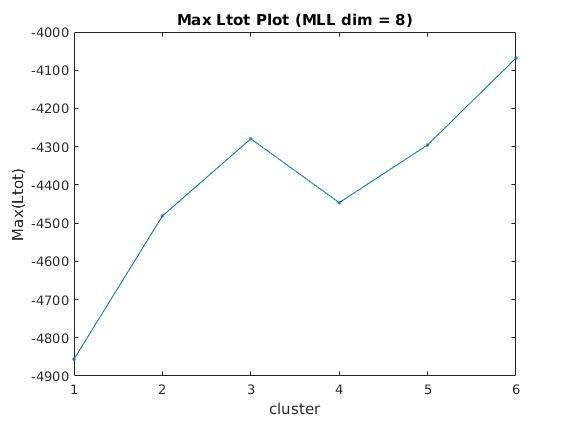


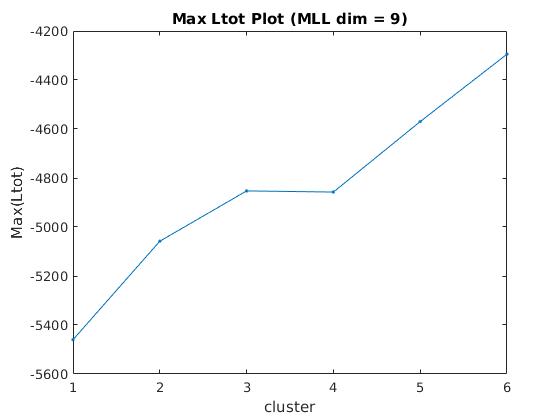


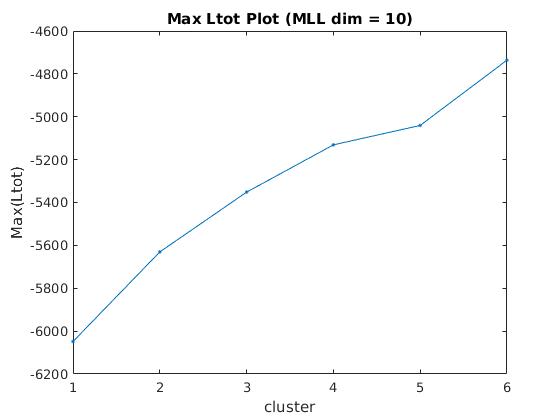


**ALL subtype dataset**


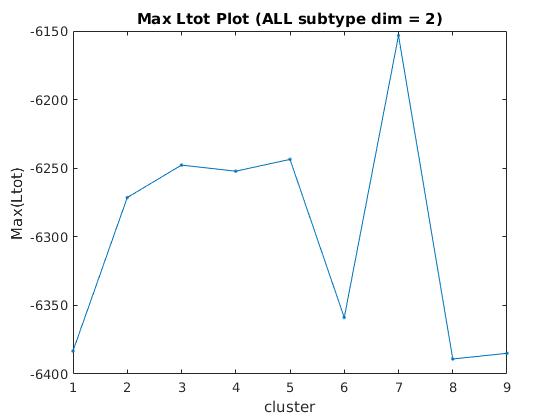


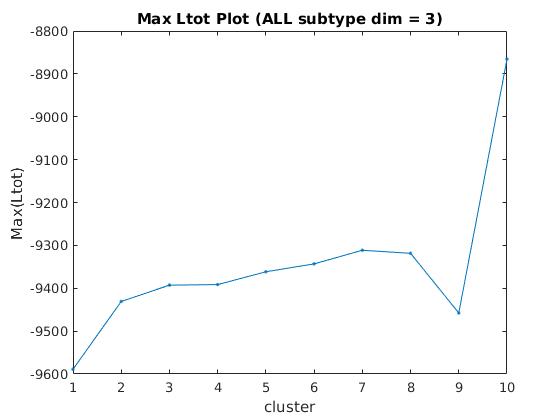


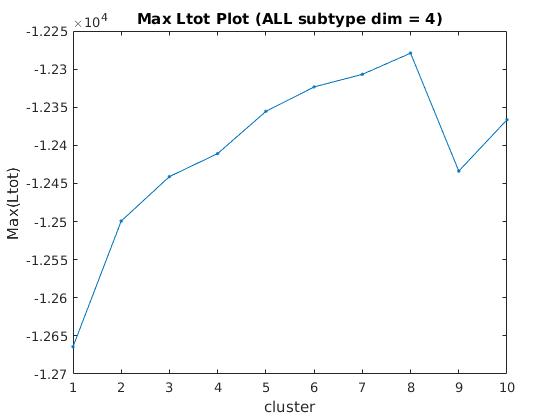


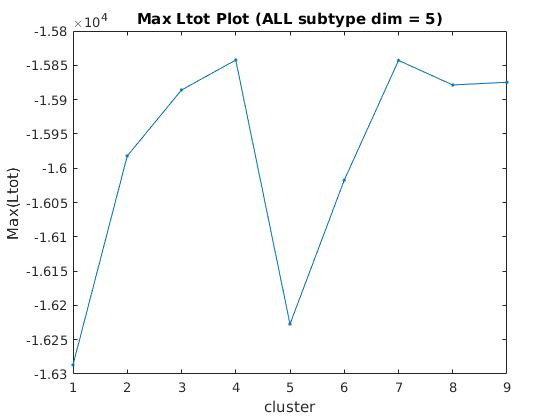


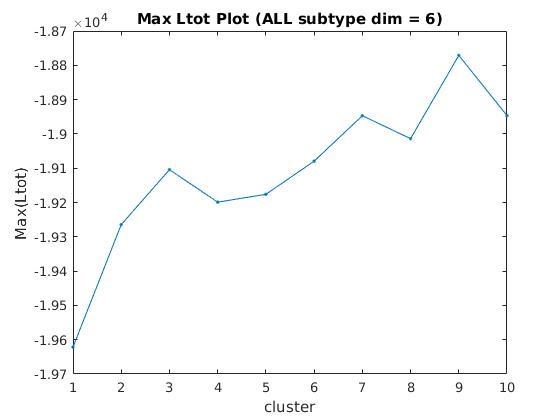


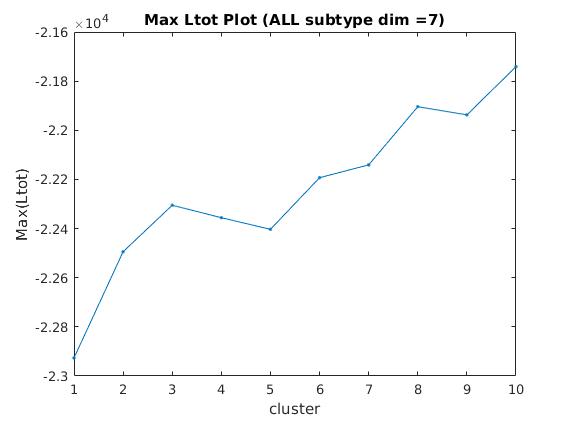


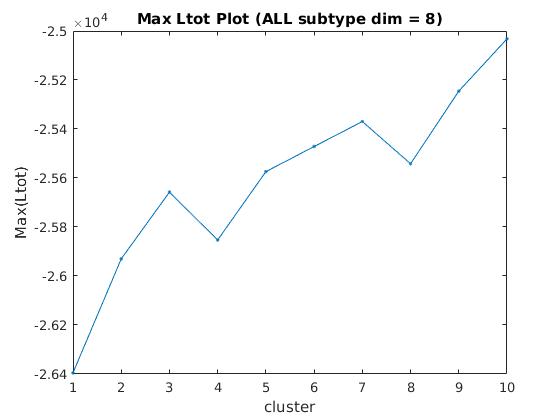

Supplement: Additional file 1: — Estimation of number of clusters using SIML method. (DOCX 408 kb) [file 12859_2016_1184_MOESM1_ESM.docx]
